# Prevalence and Influencing Factors of Depression Self-Management Among Chinese Community Residents: A Cross-Sectional Study

**DOI:** 10.3389/fpsyt.2021.559844

**Published:** 2021-05-07

**Authors:** Shuo Liu, Bing Xiang Yang, Xuan Gong, Jie Chen, Zhongchun Liu, Jun Zhang, Xiao Qin Wang

**Affiliations:** ^1^School of Health Sciences, Wuhan University, Wuhan, China; ^2^Department of Psychiatry, Renmin Hospital of Wuhan University, Wuhan, China; ^3^School of Nursing, University of Connecticut, Storrs, CT, United States

**Keywords:** depression, self-management, stigma, self-help seeking, Chinese residents

## Abstract

**Objective:** This study aimed to investigate the current status of depression self-management (DSM), and to identify influencing factors of DSM among Chinese community residents.

**Methods:** Stratified random sampling methodology was adopted in this cross-sectional survey. Respondents completed a collection of self-administered questionnaires

**Results:** The majority of participants were female (72.2%), having a mean age of 39 years (SD = 17.3). The total mean score on the DSSM was low (31.63 ± 4.69). Using multiple linear regression analysis, age ranging from 25 to 64 years old (Beta = −0.176, *p* = 0.008), having personal stigma (Beta = −0.143, *p* = 0.020) and perceived stigma (Beta = 0.127, *p* = 0.037), and having a nuclear family structure (Beta = −0.313, *p* = 0.046), good family function (Beta = 0.278, *p* < 0.001) and good help-seeking attitude (Beta = 0.159, *p* = 0.008) were associated with DSSM-knowledge. Older age (≥65 years) (Beta = −0.152, *p* = 0.034), higher CES-D scores (Beta = −0.162, *p* = 0.005), having a father with a bachelor's degree or higher level of education (Beta = −0.134, *p* = 0.047), being female (Beta = 0.147, *p* = 0.012), indicating a religious preference (Beta = 0.145, *p* = 0.017) and having good family function (Beta = 0.247, *p* = 0.001) were significantly associated with DSSM-activities.

**Conclusions:** Reducing stigma related to depression and enhancing help-seeking attitudes may be potential strategies for managing depressive symptoms among Chinese community residents.

## Introduction

More than 300 million people world-wide have experienced depression, and close to 0.8 million people die each year due to depression-related suicide (1). The prevalence of depression among Chinese community residents is 6.9% (2), and only a few obtain get timely and effective treatment. The high morbidity, high disability and high suicide rates of depression result in great social burden (3). Considering the recurrent episodes and epidemiology, depression not only requires medication, but also requires self-management to prevent (4) and reduce relapse. Self-management (SM) may be a promising approach (5); it can reduce the cost of health services (6), and offer benefits to the health care system and society (7).

The concept of SM originated with a chronic disease self-management course (CDSMC) (8), and has been applied in a variety of fields related to health. “Self-management is about the methods, skills, and strategies we use to effectively manage our own activities toward achieving certain objectives” (9). It encompasses different dimensions, such as medication management (5, 10), symptom management, informational support, change in lifestyle, social support (10); role management and emotional management (5). It offers people alternatives and helps them to maintain longer-lasting health while reducing the risk of mental health deterioration (11).

Self-management has been shown to be effective among persons with chronic depression (12). Studies reported that SM can result in positive health outcomes (8). SM may relieve or prevent the occurrence of depressive symptoms among elderly adults (13), postpartum women (14) and persons with chronic illness comorbid depression (15–17). In addition to preventing depressive symptoms, SM of depression can increase an individual's self-efficacy and facilitate their quality of life (12).

At present, SM has been increasingly applied to the prevention of depression (18). However, Studies have focused on SM of clients diagnosed with depression (16, 17), whereas very limited studies investigated the level of depression self-management (DSM) among community residents. This results in the need to understand the status of DSM in community residents, such as their level of knowledge and activities being used.

There are a variety of factors that affect DSM, the key ones include stigma (19), family function (20, 21) and help-seeking attitude. As one of the important influencing factors of SM, stigma makes people feel different from others, which becomes a barrier for people to manage themselves (19). In addition, family members play an indispensable role in the achievement of DSM in many ways, such as medication reminders, getting individuals to appointments for medical treatment (20), providing emotional support (21), and improving an individual's self-confidence and actions (22). Another influence on SM is help-seeking attitude. An individual's effective DSM relies on timely and appropriate diagnosis and treatment of depression (23). These factors have a proven impact on DSM. However, there a paucity of research exploring the impact of these variables simultaneously on DSM among Chinese community residents.

This study aimed to investigate the current status of DSM, and to identify the barriers and facilitators to DSM among Chinese community residents. Findings of this study can assist in the development of DSM strategies, and enhance the quality of life of community residents with depressive symptoms. It is anticipated that stigma, family function, help-seeking attitudes and several socio-demographic factors have significant impact on DSM.

## Methods

### Study Design and Procedure

**Figure 1:**
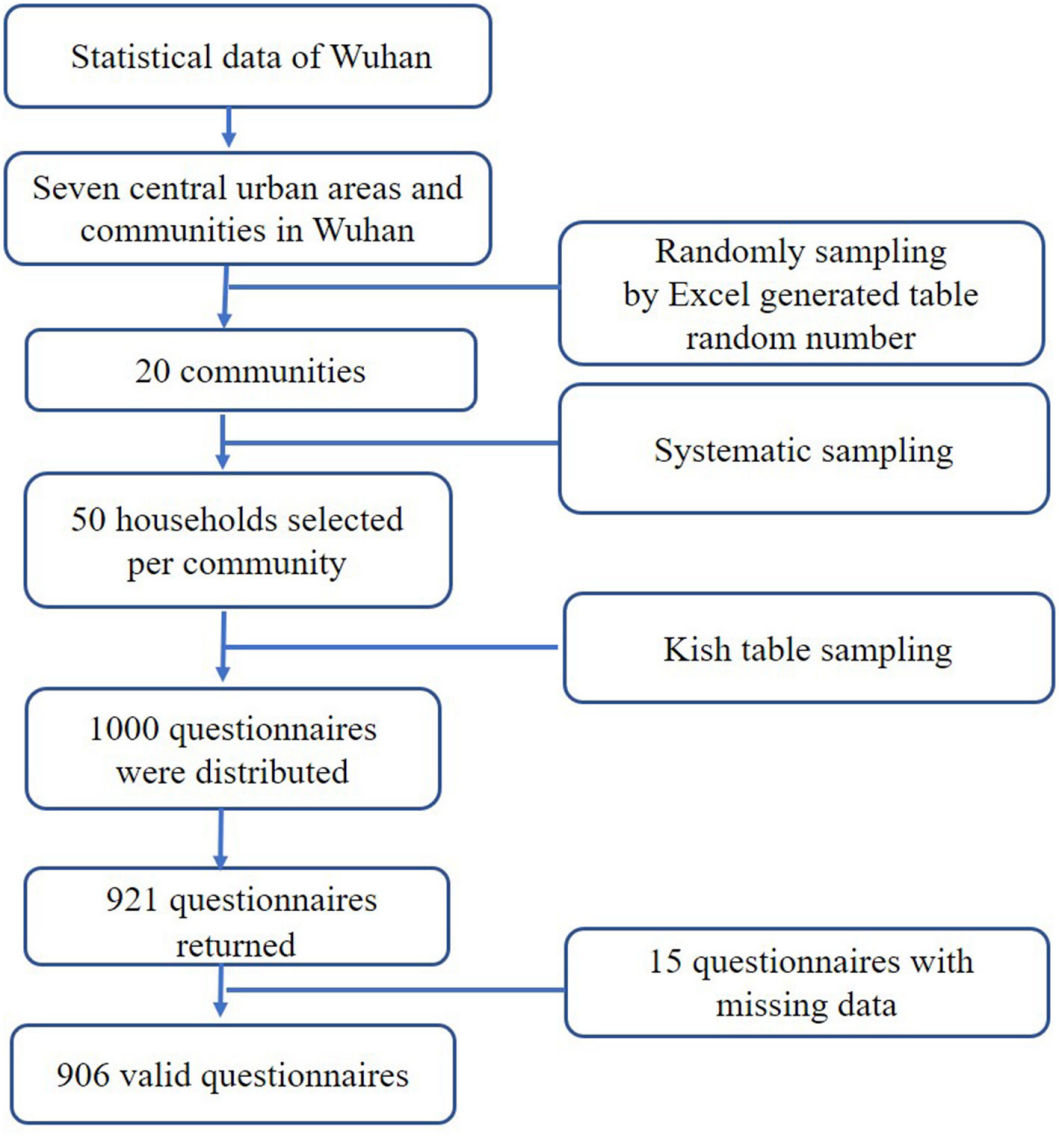
Sampling procedure.

This research was a cross-sectional study which conducted in Wuhan, the capital city of Hubei Province in central China. With a population of about 10.7 million, Wuhan is considered to have an average economic base (24). Data were obtained from seven districts and communities. Stratified random sampling was used throughout the sampling process in the household study to select the target communities, households, and individuals. This sampling method ensured that individuals in the target population who met the requirements were equally likely to be included in the study (see Figure 1). Individuals from each family were selected using Kish table sampling method, and one of eight codes (A, B1, B2, C, D, E1, E2, F) was used to target households; each family member was coded based on the family registration form (including name, age, gender, member number).

### Study Sample

The inclusion criteria were: age ≥ 15 years, and a minimum of primary school education and informed consent. Exclusion criteria were: severe physical illness; psychosis and related intellectual disability, dementia and mental disorders caused by abuse of psychoactive substances; and, severe cognitive impairment. These illnesses were screened in a review of medical records and confirmed by treating physicians. This research is part of a large cross-sectional survey (25), which recruited 2,000 samples. Due to limited grant funding, only some core indicators were included in the investigation of all 40 communities. We randomly selected 20 communities and investigated self-management of depression and other indicators. Therefore, the sample size of the current study is 1,000. In this study, G^*^power 3.1 was used to calculate the sample size based on pretest study results (Effect size = 0.22, α = 0.05, 1-β = 0.90), and the minimal sample size was 844. So, 1,000 participants meet the minimum sample size. A total of 1,000 questionnaires were distributed in this study; 921 questionnaires were returned (response rate: 92.1%) and 906 questionnaires were determined to be valid (effective callback rate: 90.6%).

### Measures

#### Demographic Information

Demographic data was obtained from participants including gender, age, ethnicity, religious preference, education, father's education, mother's education, spouse's education, employment status, occupation, marital status, household income and family structure.

#### Depression-Specific Self-Management Questionnaire (DSSM)

Depression self-management of participants was self-reported using the Depression-Specific Self-Management Questionnaire (DSSM) which was developed by Ludman et al. (26) and later modified by Gensichen et al. (27). The DSSM consists of nine items with two subscales: the first four items evaluate the respondents' specific knowledge of depression, referred to as depression self-management knowledge (DSSM 1); and the following five items evaluate respondents' specific activities for improving depressive symptoms, referred to as depression self-management activities (DSSM 2). Each subscale uses a 5-point Likert scale, ranging from 1 (strongly disagree) to 5 (strongly agree), or from 1 (never) to 5 (daily) (27). Total scores for DSSM 1 and DSSM 2 are calculated by the sum of each subscale item score, with a higher score representing greater DSSM 1 and DSSM 2. The total score is the sum of all items, ranging from nine to 45, the higher score indicates better self-management of depression. The DSSM, DSSM 1 and DSSM2 utilize 36, 16, and 20 as the critical values, respectively. The Cronbach's alpha coefficient of the DSSM was 0.725.

#### Center for Epidemiological Studies Depression Scale (CES-D)

The Center for Epidemiological Studies Depression Scale (CES-D) (28), a self-reported instrument, was used to assess participants' weekly depressive symptoms. It includes 20 items, and each item uses a 4-point scale ranging from 0 (no or hardly) to 3 (almost always) (29). The total score is 0 to 60. The higher the score, the more severe the depressive symptoms. A score of 15 or less is classified as no depressive symptoms in the past week; 16–19 indicates there may be depressive symptoms in the past week; and, a score of 20 or above indicates positive depressive symptoms in the last week. The Cronbach's α of the Chinese version was 0.9 (30). In the current study, the Cronbach's alpha coefficient was 0.93.

#### Family APGAR (Family Function)

Self-reported individual's satisfaction with family functioning was tested by the Family APGAR (31). It uses a 3-point Likert scale ranging from 0 (hardly ever) to 2 (almost always). The total score ranges from 0 to 10. The higher the score, the better the family function. More specifically, 0 to 3 represents severe family dysfunction, 4 to 6 represents moderate family dysfunction and 7 to 10 represents good family function. The Chinese version of the Family APGAR had a Cronbach's α of 0.86 (32). In the current study, Cronbach's α was 0.89.

#### Depression Stigma Scale (DSS)

The self-reported Depression Stigma Scale (DSS) (33, 34) is mainly used to investigate respondents' stigma toward depression. The DSS has 18 items with two subscales: DSS-Personal (a measure of the respondent's personal attitudes toward depression) and DSS-Perceived (a measure of the respondent's beliefs about the stigmatizing attitudes of others) (33). Each subscale consists of nine items using a 5-point Likert scale, ranging from 4 (strongly agree) to 0 (strongly disagree). In the Chinese version, the retest reliability for the DSS-Personal and DSS-Perceived scales were 0.90 and 0.73. The Cronbach's α for DSS-personal stigma and DSS-perceived stigma were 0.71 and 0.81, respectively.

#### Attitudes Toward Seeking Professional Psychological Help Scale-Short Form (ATSPPH-SF)

The self-reported ATSPPH-SF (35, 36) is mainly used to identify an individual's attitude toward seeking professional assistance. The scale has a total of 10 items using a 4-point Likert scale. The total score is 0-30 points. The higher the score, the better the help-seeking attitude. The scale has two dimensions: Openness to seeking treatment for emotional problems; Value and need in seeking treatment. The Chinese version had good reliability. Cronbach's alpha for the two dimensions were 0.757 and 0.643.

### Data Collection

Permission for this study was obtained from the Institution Review Board of Wuhan University School of Medicine (identifier: 2019YF2032). All participants were provided with informed consent and participation was considered voluntary. Respondents could withdraw at any time without prejudice. Informed consent was obtained from the guardian of participants under the age of 18. Face-to-face interview was used by the investigators who were nursing professionals and uniformly trained. Each participant received a gift after completing the questionnaire. All completed questionnaires were considered confidential and stored in a secure location accessed only by the authors. Data collection occurred from January to December 2017.

### Data Analysis

Data analysis was conducted using SPSS21.0 statistical software and a *p* value of 0.05 was considered statistically significant. Demographic characteristics were described by frequency, mean and standard deviation. The sample are tested by normality and kurtosis, showing a normal distribution. Correlation coefficient was used to analyze the correlation of continuous variables. Multiple linear regression analysis was used for analysis of influencing factors affecting DSM.

## Results

### Descriptive Data

A total of 906 residents participated in the survey and the majority were female (72.2%) having a mean age of 39 years (SD = 17.3). In addition, the mean score of the CES-D was (12.0 ± 7.97). The mean personal stigma score of participants was (19.22 ± 5.05) and the mean perceived stigma score was (21.95 ± 5.03). The average score of help-seeking attitude on “openness” and “value and need” were 10.05 (SD = 3.74), 8.09 (SD = 3.52), respectively. Details of subjects' characteristics are shown in Table 1.

**Table 1:** Demographic characteristics (*N* = 906).

**Variables**	***n***	**%**
**Gender**		
Male	251	27.8
Female	653	72.2
**Age (years)**		
16–24	199	22.1
25–64	595	66.1
≥65	106	11.8
**Ethnicity**		
Han	876	96.9
Minority	28	3.1
**Religious preference**		
No	859	95.1
Yes	44	4.9
**Education**		
Less than junior high school	176	19.4
High school/ college	398	43.9
Bachelor's degree or higher	332	36.6
**Father's education**		
Less than junior high school	464	52.6
High school/ college	326	37.0
Bachelor's degree or higher	92	10.4
**Mother's education**		
Less than junior high school	560	63.6
High school/ college	261	29.7
Bachelor's degree or higher	59	6.7
**Spouse's education**		
Less than junior high school	170	27.3
High school/ college	266	42.7
Bachelor's degree or higher	187	30.0
**Employment status**		
Unemployed/laid-off/retired	294	32.6
Full time/part time	609	67.4
**Occupation**		
Skilled worker/farmer/ business man/other	308	36.2
General company/state-owned enterprise or public institution staff/civil servant	391	45.9
Student	152	16.8
**Marital status**		
Single/separated/divorced/widowed/other	317	35.1
Cohabiting/married/remarried	584	64.9
**Depressive symptoms (CES-D score)**		
≤15	641	70.8
16~19	120	13.2
≥20	144	16.0
**Family APGAR score**		
Severe family dysfunction	123	13.7
Moderate family dysfunction	181	20.2
Good family function	592	66.1
**Monthly income level (RMB)**		
Low (<800)	29	6.4
Medium (801~3000)	284	62.7
High (≥3001)	140	30.9
**Family structure**		
Living alone	43	4.8
Nuclear family	443	49.4
Non-nuclear family	411	45.8

### Scores of DSSM

Table 2 shows the current depression management strategies used by participants. The total mean score on the DSSM was <36 (31.63 ± 4.69). The score on DSSM 1 was (14.13 ± 2.35), and the score on DSSM 2 was (17.49 ± 3.23). The highest score (4.05 ± 0.79) was found related to participation in enjoyable activities (item # 5), followed by the use of medications (item # 1, 3.75 ± 0.88). The lowest score (3.05 ± 1.07) was related to item # 8 on the frequency of participation in enjoyable activities, and item # 9 which focused on time set aside for these activities (3.07 ± 1.07).

**Table 2:** Mean scores of Depression-Specific Self-Management Questionnaire.

	**Mean**	**SD**	**Max**	**Min**
1. Some medicines are effective in the treatment of depression.	3.75	0.88	5	1
2. Over time and for most people, the side effects of antidepressants recede or can be treated.	3.38	0.88	5	1
3. If I have a personal or emotional problem I know how and where I can find assistance.	3.69	0.90	5	1
4. I can recognize the signs of a depression.	3.32	0.97	5	1
5. I can contribute toward making myself feel better by taking part in enjoyable activities.	4.05	0.79	5	1
6. I know what I have to do if I notice a deterioration in my situation or if my depressive ailment recurs.	3.66	0.90	5	1
7. I can avoid difficult situations that may trigger my depression.	3.67	0.89	5	1
8. How often have you tried to undertake enjoyable or fulfilling activities over the last month?	3.05	1.07	5	1
9. How much time have you set aside for satisfying, important, relaxing, enjoyable or pleasant activities over the last month?	3.07	1.07	5	1
Depression-specific self-management	31.63	4.69	45.00	9.00
Depression self-management-knowledge*	14.13	2.35	20.00	4.00
Depression self-management-activities**	17.49	3.23	25.00	5.00

* *Mean<16, depression self-management knowledge is low; Mean≥16, depression self-management knowledge is high*.

** *Mean<20, depression self-management activities are low; Mean≥20, depression self-management activities are high*.

### Correlation Analysis Between DSSM and Stigma, Help-Seeking Attitudes, CES-D and Family APGAR

Correlation analysis indicated that both personal stigma and the CES-D score had a negative correlation with DSSM 1 (Pearson *r* = −0.15, *p* ≤ 0.001; Pearson *r* = −0.14, *p* ≤ 0.001) which is significant (see Table 3). Openness to seeking treatment for emotional problems (*r* = 0.20) and the value of and need for seeking treatment (*r* = 0.08) was positively correlated with DSSM 1. Scores on the CES-D (*r* = −0.32) and Family APGAR (*r* = 0.15) were significantly correlated with DSSM 2.

**Table 3:** Correlation between key study variables.

	**DSSM1-Knowledge**	**DSSM2-Activities**	**DSS1**	**DSS2**	**ATSPPH-SF1**	**ATSPPH-SF2**	**CES-D**
DSSM1-Knowedge							
DSSM2-Activities	0.39**						
Stigma (DSS)							
1.Personal stigma	−0.15**	−0.06					
2.Perceived stigma	−0.03	0.01	0.55**				
Help-seeking attitude (ATSPPH-SF)							
1. Openness to seeking treatment for emotional problems	0.20**	0.02	−0.12**	−0.04			
2. Value of and need for seeking treatment	0.08*	−0.02	−0.31**	−0.22**	0.19**		
Depressive symptoms (CES-D)	−0.14**	−0.32**	−0.07*	−0.08*	0.01	−0.03	
Family function (Family APGAR)	0.16**	0.15**	0.07*	0.08*	0.10**	0.03	−0.26**

*ATSPPH-SF, Attitudes Toward Seeking Professional Psychological Help Scale-Short Form; CES-D, Center for Epidemiological Studies Depression Scale; DSS, Depression Stigma Scale; DSSM, Depression-Specific Self-Management; Family APGAR, family function scale*.

* *p < 0.05*;

** *p < 0.01*.

### Influencing Factors on DSM

Using multiple linear regression analysis, influencing factors of DSM are shown in Table 4. The multiple linear regression models were statistically significant, F_DSSM−knowledge_ = 3.039, *p* < 0.001, with a R-square value (R^2^) of 0.505; F_DSSM−activities_ = 2.980, *p* < 0.001, R^2^ = 0.251. Findings indicate that age ranging from 25 to 64 years old (Beta = −0.176, *p* = 0.008), having personal stigma (Beta = −0.143, *p* = 0.020), and being in a nuclear family structure (Beta = −0.313, *p* = 0.046) were negatively associated with DSSM 1, while good family function (Beta = 0.278, *p* < 0.001) and good help-seeking attitude (Beta = 0.159, *p* = 0.008) were associated in a positive direction with DSSM 1. Older age (≥65 years) (Beta = −0.152, *p* = 0.034), higher CES-D scores (Beta = −0.162, *p* = 0.005), and father's bachelor or higher education level (Beta = −0.134, *p* = 0.047) were negatively associated with DSSM 2, while individuals being female (Beta = 0.147, *p* = 0.012), having a religious preference (Beta = 0.145, *p* = 0.017), and having good family function (Beta = 0.247, *p* = 0.001) were positively associated with DSSM 2.

**Table 4:** Multiple linear regression of influencing factors of depression self-management.

	**DSSM 1**				**DSSM 2**			
	**B**	**Beta**	***t***	***p***	**B**	**Beta**	***t***	***p***
Gender (male=0, female=1)	0.305	0.049	0.836	0.404	1.305	0.147	2.524	0.012*
Age (15~24=0)	—	—	—	—	—	—	—	—
Age (25~64=1)	−5.266	−0.176	−2.661	0.008*	−4.658	−0.110	−1.659	0.098
Age (≥65 =2)	−0.413	−0.072	−1.007	0.315	−1.238	−0.152	−2.127	0.034*
Religious preference (none=0, yes=1)	0.540	0.060	1.003	0.317	1.839	0.145	2.409	0.017*
Father's education (less than junior high school=0)	—	—	—	—	—	—	—	—
Father's education (high school/ college=1)	0.717	0.132	1.643	0.102	0.313	0.041	0.506	0.641
Father's education (bachelor's degree or higher=2)	−1.243	−0.115	−1.721	0.086	−2.044	−0.134	−1.996	0.047*
Family function (severe family dysfunction=0)	—	—	—	—	—	—	—	—
Family function (moderate family dysfunction=1)	0.880	0.129	1.797	0.073	0.722	0.075	1.040	0.299
Family function (good family function=2)	1.464	0.278	3.732	<0.001*	1.834	0.247	3.297	0.001*
CES-D score (≤15)	—	—	—	—	—	—	—	—
CES-D score (16~19)	−0.576	−0.055	−0.951	0.342	−2.412	−0.162	−2.808	0.005*
CES-D score (≥20)	0.083	0.129	1.797	0.073	−2.425	−0.226	−3.865	<0.001*
Stigma								
Personal stigma (none=0, yes=1)	−0.770	−0.143	−2.343	0.020*	−0.603	−0.079	−1.294	0.197
Perceived stigma (none=0, yes=1)	0.772	0.127	2.102	0.037*	0.490	0.057	0.940	0.384
Help-seeking attitude								
Openness to seeking treatment for emotional problems (none=0, yes=1)	0.826	0.159	2.685	0.008*	0.060	0.008	0.138	0.891
Family structure (living alone=0)	—	—	—	—	—	—	—	—
Family structure (nuclear family=1)	−1.580	−0.313	−2.003	0.046*	1.449	0.203	1.296	0.196
Family structure (non-nuclear family=2)	−1.373	−0.265	−1.769	0.078	1.093	0.149	0.993	0.322

*CES-D, Center for Epidemiological Studies Depression Scale; DSSM, Depression-Specific Self-Management*.

* *p < 0.05*.

## Discussion

This study found that the level of DSSM was negative in Chinese community residents. Current study results are consistent with previous studies in Rawalpindi (37), Australia (38) with self-management knowledge on depression being negative among community residents in China. Globally, it appears that depression-related knowledge is lacking in the general population. In addition, it also reflects that the general population pays less attention to depression in China. This reinforces the need for healthcare providers to popularize the importance of knowledge about depression. Self-management activities on depression were also negative, which was another interesting finding of this study. Previous study showed that engaging in a variety of activities that suitable for ourselves contribute to better self-management (39). However, it is difficult to choose effective individualized self-management strategies use them consistently. The Internet and other electronic media resources allow for greater access to relevant information, but individuals may lack the initiative to utilize this information. In the face of avoiding complex interpersonal relationships, many people adopt a more solitary lifestyle, and this could contribute to this lack of initiative in engaging in self-management activities.

This study found that good family function was positively associated with DSM knowledge. This finding implied that family function and/or family support played an important role in DSM, which is consistent with a study by Polacsek (23). Family provides a supportive atmosphere for self-management of chronic illness (including depression). For instance, family members were crucial in providing practical support for individuals with mental health problems by offering medication reminders and promoting physical activities (21). Encouraged by their family, members seek diagnosis, receive treatment and take steps to better manage themselves (23). In addition, families provide emotional support for their members, reduce isolation and enhance members' autonomy in self-management (21, 40).

Families can also be a deterrent in promoting a family member's self-management behaviors (19). The results showed that having a father with a bachelor's degree or higher level of education was negatively associated with DSM activities. A family's lack of recognition of a member's illness and their prejudicial viewpoint against individuals with depression can aggravate the member's shame and hinder their SM (41, 42). Having a father with a higher level of education might lead to the father being focused on his occupational and social position, which results in less attention on family life, family members and their mental health. Thus, there may be an unwillingness on the part of the father to accept a family member having depression and helping to promote better strategies for self-management. Moreover, overprotection by the family of their member promotes low confidence in the individual's abilities and thus negatively affects their self-management strategies (43). The health problems of other family members can have competitive demands, and individuals are unable to focus on their own management (20).

Having a positive help-seeking attitude was also an important factor of DSM knowledge. Participants who were more open to seeking help (such as psychotherapy and psychological counseling) during an emotional crisis were noted to have better DSM knowledge. Having access to the health care system can also improve self-management abilities (23). Being willing to seek assistance and receiving practical guidance and feedback on SM from health care professionals promotes better self-management abilities (20).

Positively associated with DSM activities were included being female and having a religious preference. Women are more likely to participate in activities to improve depressive symptoms than men which is consistent with a previous study of persons having depression (44). An interesting finding in this study was that having a religious preference had a significant impact on DSM activities. Similarly, studies by Park (45) showed that individuals who had religious beliefs are more likely to manage depressive symptoms effectively. Religion not only provides spiritual support for individuals, but also offers an opportunity for the individual to have a helpful relationship with others who share their religious beliefs. This form of support plays a solid role in a person's self-management abilities (46).

More depressive symptoms were negatively associated with DSM activities. This is supported by other research that has shown that people were less likely to access self-management information when experiencing severe depressive symptoms (47). A probable explanation is that an individual with major depressive disorder experiences symptoms such as reduced energy (48), lack of interest (49) and motivation (46), which may hinder their effective use of enjoyable activities (50). Cognition may be distorted and the individual can experience a sense of shame that hinders self-management. And, in turn, DSM could improve depressive symptoms (11). Providing support to people with severe depression increases their self-efficacy and enhances self-management behaviors (11, 27).

In the current study, community residents who had higher perceived stigma were more likely to have better knowledge, and people who had higher personal stigma were less likely to have better self-management knowledge. This is supported by another study which found that stigma (perceived stigma) was a motivator of self-management (20). Research has shown that in order to avoid shame, individuals who seek to improve their knowledge of depression and mental health literacy also enhance self-management abilities (20, 23). However, stigma (personal stigma) was also reported to have a negative impact on self-management abilities (23). Stigma prevents respondents from receiving any information about depression, and lack of knowledge of DSM was a barrier to conducting self-management behaviors (19).

Stigma and help-seeking attitude were significantly correlated. Stigma may be one of the barriers to seeking help (51, 52). Participants who had higher stigma were less reluctant to seek help (23), in addition, some non-mental health professionals had higher stigma toward mental disorders and want to keep distance from such people (53), which hindered their ability to meet personal needs. People who are open to seeking help have access to health care resources to develop their health-related literacy, thereby improving their self-management abilities (54) and reducing stigma (52). In addition, both stigma and help-seeking attitude have proven to have a significant impact on DSM. Therefore, these factors should be taken into account when providing effective strategies or interventions to promote self-management and prevent depression.

## Study Limitations and Implications

Limitations to this research study have been identified. This was a cross-sectional survey, which could not explain the causal relationship between influencing factors and dependent variables. In addition, the nature of voluntary participation may lead to potential selection bias. This study was done in central China, which limits generalization of findings to other geographic regions. There was a gender imbalance in the sample, the gender imbalance in the sample may influence the representative of the population. While there are limitations, the findings have important implications for future research and practice: it was found that individuals with severe depressive symptoms were less likely to engage in DSM which indicates that greater efforts should be placed on early screening and intervention. In addition, health care providers could be encouraged to address the issue of stigma. This can include educating community residents about depression by providing handouts and educational materials such as videos, flyers and posters at community health centers. To address the issue of improving help-seeking attitude related to DSM, more attention needs to be paid to the use of motivational interviewing to enhance awareness of and the advantages of DSM. Further research could explore the mediating effect between the variables included in this study and the DSSM.

## Conclusions

Findings of this study indicate that Chinese community residents had a relative low level of using DSM. Reducing stigma related to depression and enhancing help-seeking attitudes may be potential strategies for managing depressive symptoms and improving the quality of life of Chinese community residents.

## Data Availability Statement

The raw data supporting the conclusions of this article will be made available by the authors, without undue reservation.

## Ethics Statement

The studies involving human participants were reviewed and approved by Institution Review Board of Wuhan University School of Medicine. The patients/participants provided their written informed consent to participate in this study.

## Author Contributions

BXY, SL, XG, and XQW designed the study and wrote the research protocol. SL, BXY, XG, JC, ZCL, JZ, and XQW did the literature review, managed the field survey, quality control, and statistical analysis and prepared the manuscript draft. XQW, JZ, BXY, and JC contributed to the revisions in depth for the manuscript. ZCL, BXY, XQW, and JC supervised the survey and checked the data. All authors contributed to and approved the final manuscript.

## Conflict of Interest

The authors declare that the research was conducted in the absence of any commercial or financial relationships that could be construed as a potential conflict of interest.
